# Documented pain phenotypes and interventional pain-care exposure in patients with dystonia: a retrospective text-mining cohort study

**DOI:** 10.1016/j.prdoa.2026.100505

**Published:** 2026-10-03

**Authors:** Shachar Zion Shemesh, Tsvia Fay-Karmon, Paz Kelmer, Jose Asprilla, Zaki Hinnawi, Itay Goor-Aryeh, Yotam Hadari, Lior Ungar

**Affiliations:** a Department of Neurosurgery, Sheba Medical Center, Ramat Gan, Israel; b Gray Faculty of Medical and Health Sciences, Tel Aviv University, Tel Aviv, Israel; c Movement Disorders Institute and Department of Neurology, Sheba Medical Center, Ramat Gan, Israel; d Pain Center, Sheba Medical Center, Ramat Gan, Israel; e Dina Recanati School of Medicine, Reichman University, Herzliya, , Israel

**Keywords:** Dystonia, Chronic pain, Cervical dystonia, Text mining, Natural language processing, Deep brain stimulation, Botulinum toxin, Interventional pain, Neuropathic pain, Central sensitization

## Abstract

**Background:**

Pain is a non-motor manifestation of dystonia, but its anatomical heterogeneity and treatment exposure are incompletely characterized. We described pain phenotypes in patients with dystonia referred to a tertiary pain service.

**Methods:**

In this retrospective cohort study, electronic health record text from 2010 to 2026 was collapsed from 108,797 raw rows to 1860 encounters among 129 patients. A Hebrew-English rule-based pipeline identified pain phenotypes and documented treatment exposures. A category-stratified random sample of 129 records was manually audited by a senior neurosurgeon blinded to pipeline output. The primary objective was phenotype distribution; treatment exposures and phenotype-treatment associations were secondary or exploratory.

**Results:**

Mean age was 67.0 years (SD 6.0), 61 patients (47.3%) were men, mean age at dystonia onset was 60 years, and all patients had at least four years of documented disease. Phenotype-codable pain was identified in 125 patients (96.9%). The most frequent phenotypes were limb/extremity (72.9%), spine/radicular (72.1%), cervico-occipital (53.5%), neuropathic-descriptor (43.4%), and myofascial/muscle pain (37.2%). At least one prespecified interventional procedure category was documented in 86 patients (66.7%). Cervical dystonia was associated with cervico-occipital pain (69.4% vs 43.8%; OR 2.91, 95% CI 1.38–6.18; *P* = .006). Treatment associations were interpreted as co-documentation patterns affected by indication bias.

**Conclusions:**

Pain in this referral-enriched cohort was anatomically and mechanistically heterogeneous and frequently accompanied by documented procedural and medication exposure. The findings support prospective assessment using validated pain instruments, but do not establish diagnoses, treatment efficacy, or population prevalence.

## Highlights

• Pain-referred dystonia patients showed heterogeneous pain phenotypes.
• Spine/radicular and limb pain were the most frequent phenotypes.
• Two-thirds had documented interventional pain treatment burden.
• Cervical dystonia was enriched for cervico-occipital pain.
• Findings support structured pain phenotyping in dystonia care.

## 1 Introduction

Dystonia is a heterogeneous movement disorder characterized by sustained or intermittent muscle contractions that produce abnormal postures, twisting movements, tremor, and task-dependent overflow. Contemporary classifications distinguish focal, segmental, multifocal, hemidystonic, and generalized distributions and consider etiology as a separate axis.[1], [2] Non-motor manifestations, including pain, mood disturbance, sleep impairment, and reduced quality of life, contribute substantially to disability [3], [4] (See Fig. 1, Fig. 2, Fig. 3, Fig. 4) (See Table 1, Table 2, Table 3, Table 4).

**Fig. 1:**
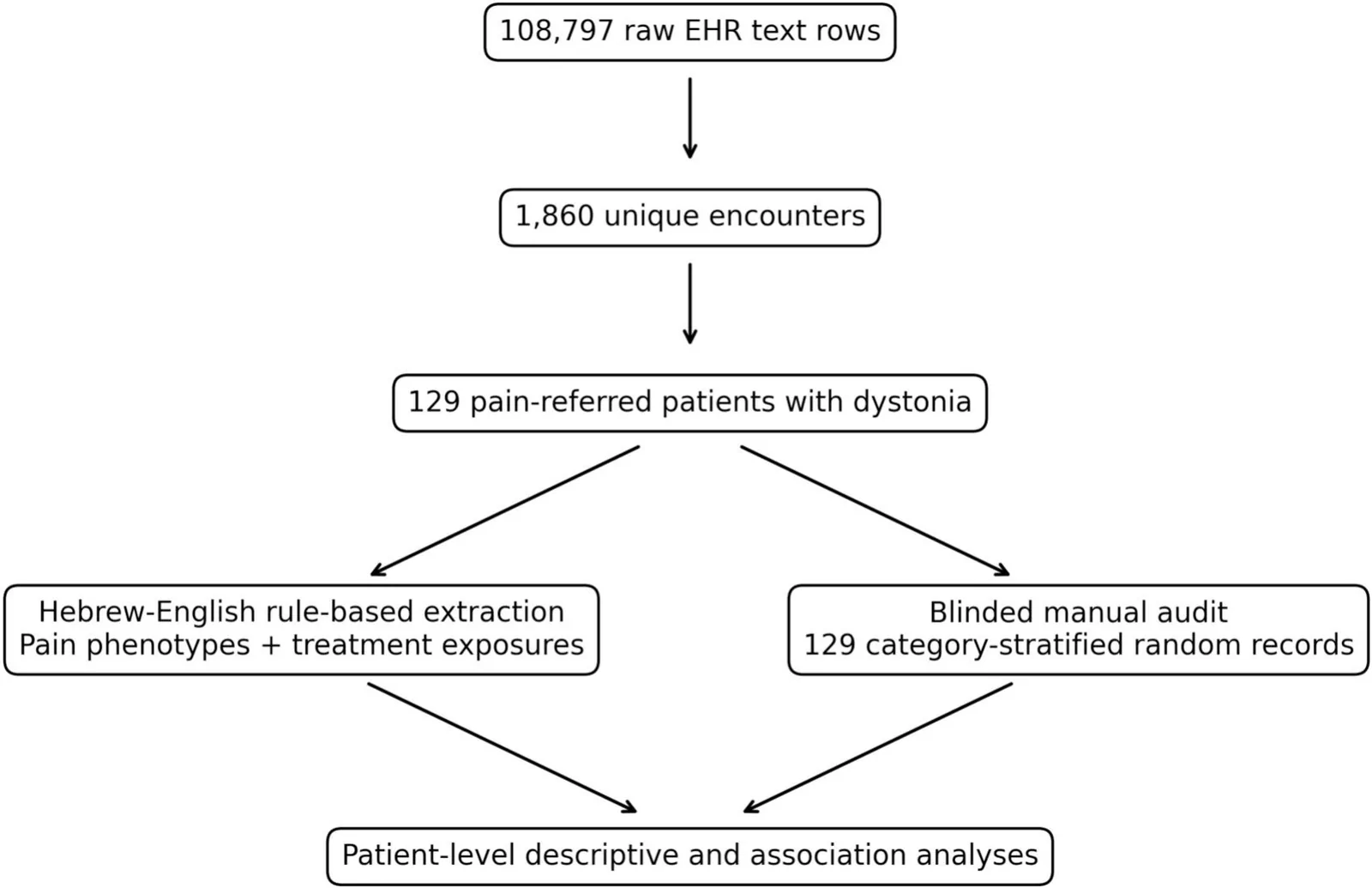
Cohort derivation and analytic workflow. Raw EHR text was collapsed to encounters and patient-level features. A blinded category-stratified manual audit was conducted independently of pipeline output.

**Fig. 2:**
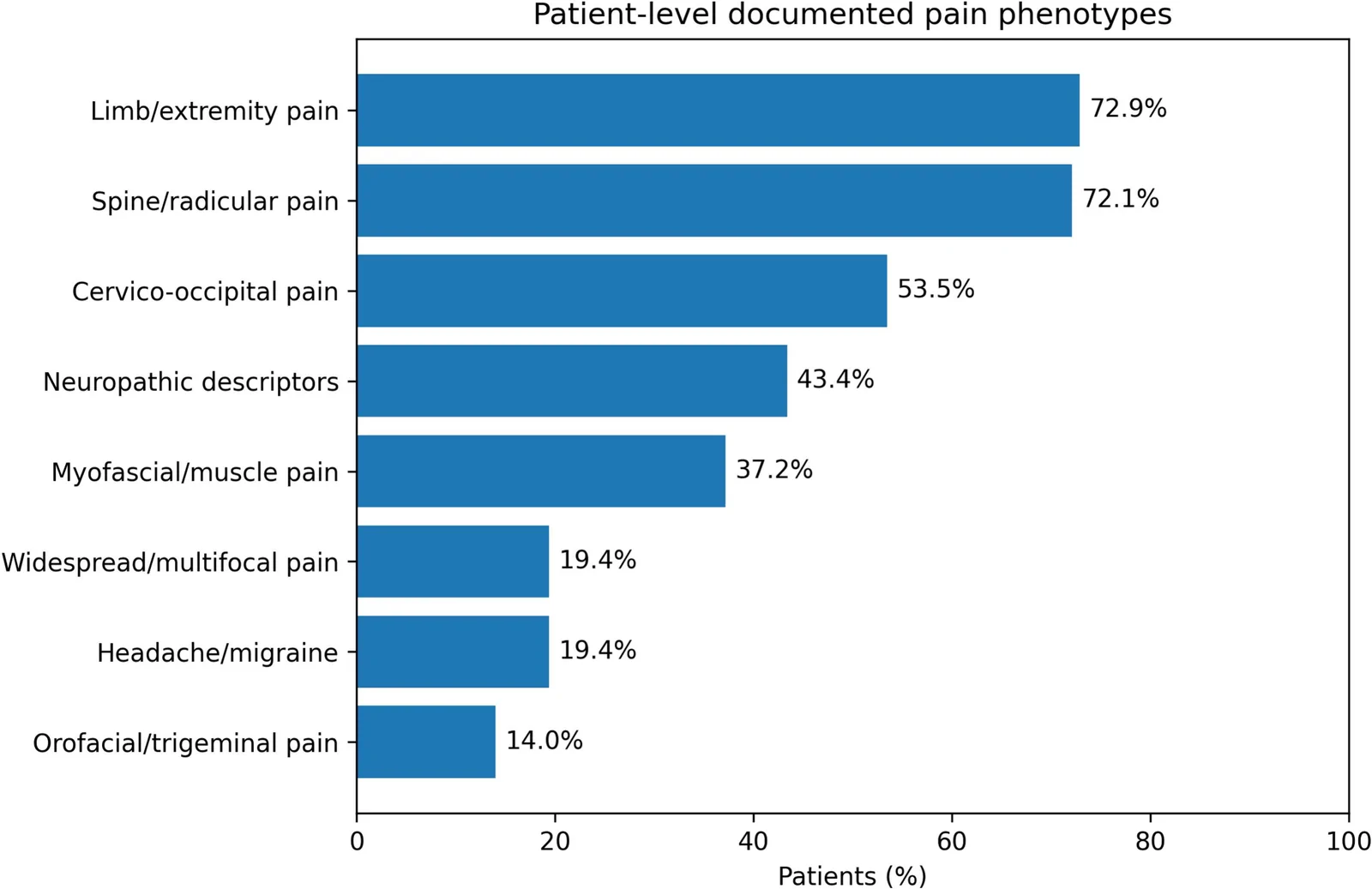
Patient-level documented pain phenotypes among pain-referred patients with dystonia. Phenotypes were non-mutually exclusive and do not estimate population prevalence.

**Fig. 3:**
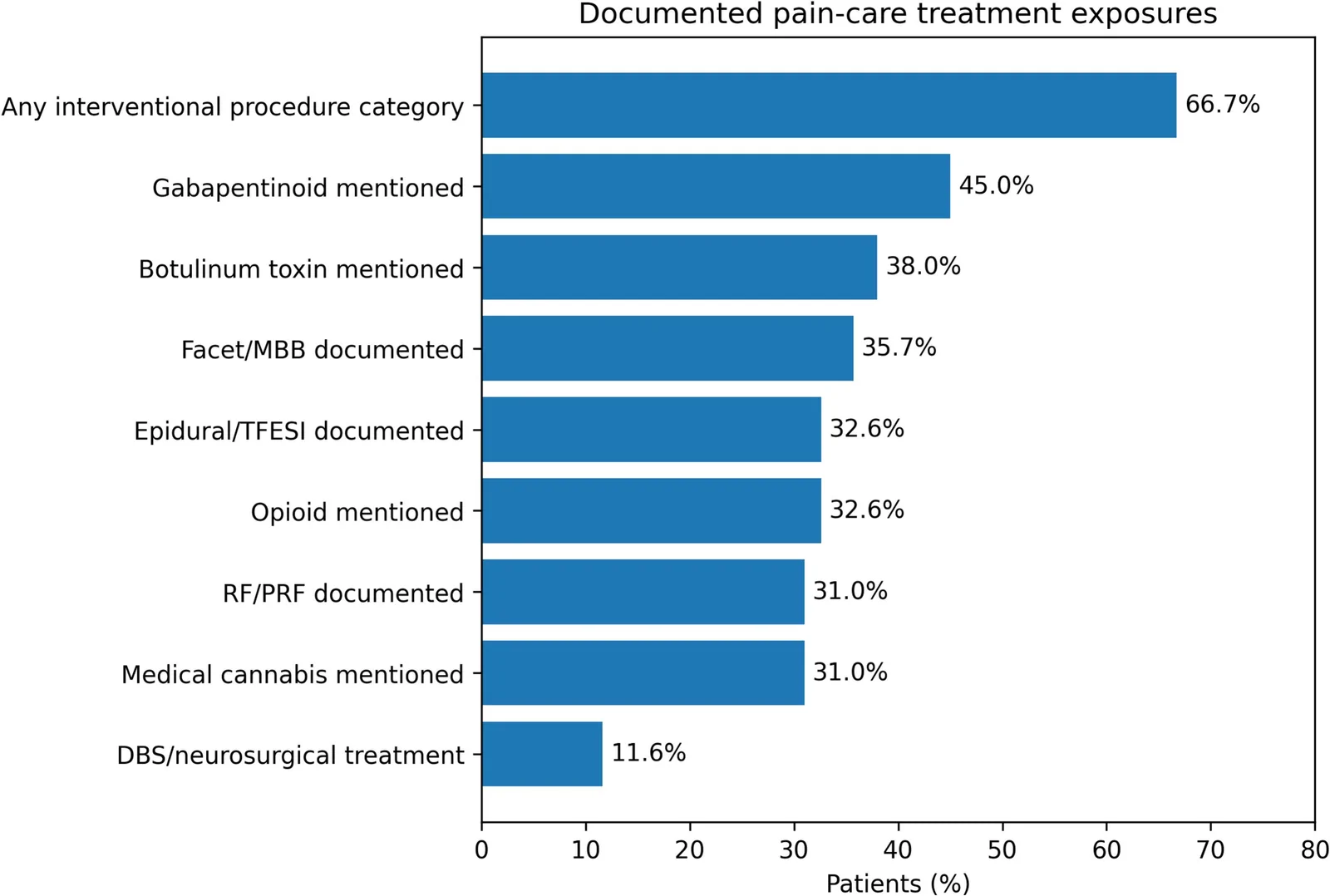
Documented pain-care treatment exposures. Flags indicate EHR documentation of administration, prescription, planning, or discussion and do not establish efficacy.

**Fig. 4:**
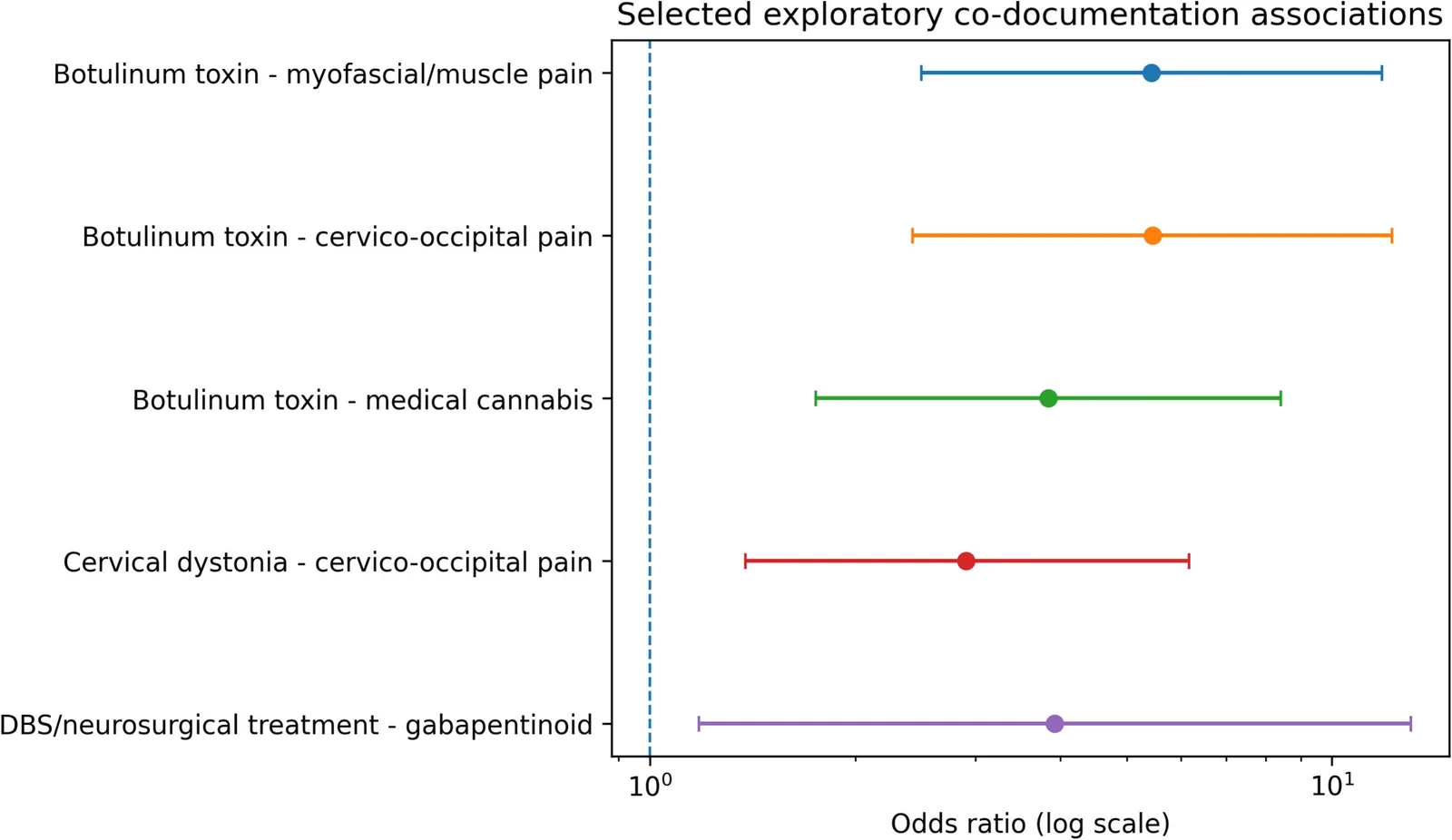
Selected patient-level co-documentation associations. Odds ratios are displayed on a logarithmic scale. Findings are unadjusted and should not be interpreted causally.

**Table 1:** Cohort characteristics and analytic structure.

**Characteristic**	**Value**
Unique patients	129
Raw EHR text rows	108,797
Unique visits/encounters	1860
Study period	October 17, 2010 to May 10, 2026
Age at cohort entry, years	67.0 (SD 6.0)
Men	61 (47.3%)
Age at dystonia onset, years	60 (mean)
Documented dystonia duration	At least 4 years in all patients
Cervical dystonia documentation	49 (38.0%)
Visits per patient	5 (IQR, 2–12)
Observed follow-up, years	0.95 (IQR, 0.06–3.87)
Manual audit sample	129 category-stratified random clinical records
Primary analytic unit	Patient-level aggregation

Values are mean +/− standard deviation, median (interquartile range), or n (%), as indicated. Disease duration refers to documented dystonia history.

**Table 2:** Patient-level documented pain phenotypes.

**Phenotype**	**Patients**	**Patient %**	**95% CI**	**Visits**	**Visit %**
Phenotype-codable pain documentation	125/129	96.9%	92.3–98.8%	1228	66.0%
Widespread/multifocal pain	25/129	19.4%	13.5–27.0%	223	12.0%
Cervico-occipital/cervicogenic pain	69/129	53.5%	44.9–61.9%	621	33.4%
Headache/migraine	25/129	19.4%	13.5–27.0%	310	16.7%
Spine/radicular pain	93/129	72.1%	63.8–79.1%	966	51.9%
Limb/extremity pain	94/129	72.9%	64.6–79.8%	877	47.2%
Orofacial/trigeminal pain	18/129	14.0%	9.0–21.0%	159	8.5%
Neuropathic descriptors	56/129	43.4%	35.2–52.0%	357	19.2%
Myofascial/muscle pain	48/129	37.2%	29.4–45.8%	254	13.7%
**Phenotype**	**Patients**	**Patient %**	**95% CI**	**Visits**	**Visit %**
Phenotype-codable pain documentation	125/129	96.9%	92.3–98.8%	1228	66.0%
Widespread/multifocal pain	25/129	19.4%	13.5–27.0%	223	12.0%
Cervico-occipital/cervicogenic pain	69/129	53.5%	44.9–61.9%	621	33.4%
Headache/migraine	25/129	19.4%	13.5–27.0%	310	16.7%
Spine/radicular pain	93/129	72.1%	63.8–79.1%	966	51.9%
Limb/extremity pain	94/129	72.9%	64.6–79.8%	877	47.2%
Orofacial/trigeminal pain	18/129	14.0%	9.0–21.0%	159	8.5%
Neuropathic descriptors	56/129	43.4%	35.2–52.0%	357	19.2%
Myofascial/muscle pain	48/129	37.2%	29.4–45.8%	254	13.7%
**Phenotype**	**Patients**	**Patient %**	**95% CI**	**Visits**	**Visit %**
Phenotype-codable pain documentation	125/129	96.9%	92.3–98.8%	1228	66.0%
Widespread/multifocal pain	25/129	19.4%	13.5–27.0%	223	12.0%
Cervico-occipital/cervicogenic pain	69/129	53.5%	44.9–61.9%	621	33.4%
Headache/migraine	25/129	19.4%	13.5–27.0%	310	16.7%
Spine/radicular pain	93/129	72.1%	63.8–79.1%	966	51.9%
Limb/extremity pain	94/129	72.9%	64.6–79.8%	877	47.2%
Orofacial/trigeminal pain	18/129	14.0%	9.0–21.0%	159	8.5%
Neuropathic descriptors	56/129	43.4%	35.2–52.0%	357	19.2%
Myofascial/muscle pain	48/129	37.2%	29.4–45.8%	254	13.7%

Phenotypes are non-mutually exclusive. Percentages use 129 pain-referred patients as the denominator and describe documentation within this referral-enriched cohort, not population prevalence.

**Table 3:** Documented treatment and procedure exposures in the electronic health record.

Documented exposure	**Patients**	**Patient %**	**Visits**
Any prespecified interventional procedure category	86/129	66.7%	–
Botulinum toxin	49/129	38.0%	216
DBS/neurosurgical dystonia treatment	15/129	11.6%	63
Occipital/GON block	14/129	10.9%	81
Epidural/TFESI	42/129	32.6%	299
Facet/MBB	46/129	35.7%	233
RF/PRF	40/129	31.0%	271
Trigger-point injection	14/129	10.9%	105
Opioid	42/129	32.6%	246
Gabapentinoid	58/129	45.0%	271
Medical cannabis	40/129	31.0%	190
Other pain medication categories	Extracted but not emphasized	–	–
Documented exposure	**Patients**	**Patient %**	**Visits**
Any prespecified interventional procedure category	86/129	66.7%	–
Botulinum toxin	49/129	38.0%	216
DBS/neurosurgical dystonia treatment	15/129	11.6%	63
Occipital/GON block	14/129	10.9%	81
Epidural/TFESI	42/129	32.6%	299
Facet/MBB	46/129	35.7%	233
RF/PRF	40/129	31.0%	271
Trigger-point injection	14/129	10.9%	105
Opioid	42/129	32.6%	246
Gabapentinoid	58/129	45.0%	271
Medical cannabis	40/129	31.0%	190
Other pain medication categories	Extracted but not emphasized	–	–

Flags indicate documented administration, prescription, planning, or discussion. They do not establish standardized exposure duration, treatment indication, response, or efficacy.

**Table 4:** Selected patient-level associations.

Exposure	Outcome	OR	95% CI	*P* value	BH q value
Botulinum toxin	Myofascial/muscle pain	5.44	2.50–11.84	<0.001	0.0012
Botulinum toxin	Cervico-occipital/cervicogenic pain	5.46	2.43–12.25	<0.001	0.0012
Botulinum toxin	Medical cannabis	3.84	1.75–8.41	0.0008	0.029
Cervical dystonia	Cervico-occipital/cervicogenic pain	2.91	1.38–6.18	0.006	0.112
DBS/neurosurgical treatment	Gabapentinoid	3.92	1.18–13.06	0.026	0.247
Cervical dystonia	Facet/MBB	2.93	1.38–6.19	0.008	0.118

Odds ratios were estimated using Fisher exact tests. The cervical dystonia comparison was prespecified; other associations were exploratory and Benjamini-Hochberg adjusted. All findings are unadjusted and subject to indication bias.

Pain is particularly prominent in cervical dystonia and may be as disabling as the motor syndrome.[5], [6], [7], [8], [9] The Dystonia-Pain Classification System (Dystonia-PCS) provides a validated prospective framework for classifying chronic pain according to its relationship to dystonia and for grading intensity, frequency, and functional impact.^5^ Real-world tertiary pain records, however, may contain overlapping cervico-occipital, myofascial, spine/radicular, neuropathic-like, headache, limb, and orofacial features that are not captured by a single diagnostic label.

Retrospective electronic health record analysis can characterize this heterogeneity and the accompanying treatment burden, but documentation patterns must not be equated with adjudicated diagnoses, treatment administration, or therapeutic efficacy. Neuropathic-like and sensitization-associated symptoms have been described in cervical dystonia, while degenerative spine disease and radiculopathy may coexist with or be aggravated by abnormal posture.[10], [11], [12], [13], [14], [15], [16], [17] These overlaps are clinically relevant to movement-disorders, neurosurgical, and interventional pain practice.

The primary objective of this study was to describe the patient-level distribution of documented pain phenotypes among patients with dystonia referred to a tertiary pain service. Secondary objectives were to describe documented interventional and medication exposures. The prespecified clinical comparison evaluated whether cervical dystonia was associated with cervico-occipital pain; all other phenotype-treatment associations were exploratory and hypothesis-generating.

## 2 Methods

### 2.1 Study design and data source

We conducted a retrospective, single-center cohort study using an institutional electronic health record extract containing structured visit metadata and Hebrew- and English-language free-text documentation for patients with dystonia referred from a movement disorders clinic to a tertiary pain service between October 17, 2010 and May 10, 2026. The referral pathway deliberately selected a pain-enriched population; therefore, the study was designed to characterize documented pain patterns within this cohort and not to estimate pain prevalence in the wider dystonia population.

### 2.2 Cohort construction and clinical variables

The source file contained 108,797 raw text rows. Repeated note sections were collapsed to 1860 unique encounters using patient identifier, medical record identifier, and visit date, and encounters were then aggregated to 129 unique patients. Analyses were primarily patient-level to avoid treating repeated note fragments as independent observations. Mean age at cohort entry was 67.0 years (SD 6.0); 61 patients (47.3%) were men. Mean age at dystonia onset was 60 years, and all patients had at least four years of documented dystonia history. Detailed etiologic subtype and standardized motor-severity measures were not consistently available in the analytic extract.

### 2.3 Pain phenotype extraction

A conservative rule-based multilingual pipeline was developed to identify clinically specific pain concepts in Hebrew and English free text. Non-mutually exclusive patient-level phenotypes included widespread/multifocal pain, cervico-occipital/cervicogenic pain, headache/migraine, spine/radicular pain, limb/extremity pain, hip/pelvis/sacroiliac pain, orofacial/trigeminal pain, neuropathic descriptors, and myofascial/muscle pain. Broad or anatomically ambiguous terms were refined or excluded when they did not imply pain. Positive flags were aggregated across all available encounters for each patient. Pipeline outputs represent documented concepts rather than independently adjudicated pain diagnoses.

### 2.4 Treatment and interventional exposure extraction

Rule-based treatment flags identified documentation of botulinum toxin, deep brain stimulation or other neurosurgical dystonia treatment, baclofen pump therapy, occipital and other nerve blocks, trigeminal procedures, epidural or transforaminal epidural steroid injections, facet or medial branch blocks, radiofrequency or pulsed radiofrequency procedures, joint injections, trigger-point injections, opioids, gabapentinoids, antidepressants used for pain, medical cannabis, non-opioid analgesics, and baclofen or tizanidine. These flags indicate that a treatment was documented as administered, prescribed, planned, or discussed; they do not establish standardized exposure duration, indication, response, or efficacy.

### 2.5 Manual audit

A category-stratified random sample of 129 clinical records, selected to include examples from every pain-phenotype category, was independently reviewed by a senior neurosurgeon who was blinded to the pipeline output. The audit was used to assess clinical face validity and identify overly broad rules before the final analysis. Because record-level true-positive, false-positive, true-negative, and false-negative matrices were not retained, formal sensitivity, specificity, positive predictive value, and inter-rater agreement could not be reconstructed. We therefore describe this procedure as a blinded manual audit rather than formal algorithm validation.

### 2.6 Study outcomes and statistical analysis

The primary outcome was the patient-level proportion with each documented pain phenotype. Secondary outcomes were patient-level proportions with documented treatment and procedure exposures. Counts and percentages were reported with Wilson 95% confidence intervals. The prespecified association between cervical dystonia and cervico-occipital pain was evaluated with Fisher exact testing and an odds ratio with 95% confidence interval. Additional phenotype-treatment co-documentation screens were exploratory, unadjusted, and corrected using the Benjamini-Hochberg procedure. No multivariable models were fitted because key clinical covariates, including standardized motor severity and complete dystonia subtype, were unavailable. Analyses were interpreted as descriptive or hypothesis-generating.

## 3 Results

### 3.1 Cohort characteristics

The final cohort comprised 129 patients and 1860 unique encounters derived from 108,797 raw EHR rows. The median number of encounters per patient was 5 (IQR, 2–12), and median observed follow-up was 0.95 years (IQR, 0.06–3.87). Mean age was 67.0 years (SD 6.0), 61 patients (47.3%) were men, mean age at dystonia onset was 60 years, and all patients had at least four years of documented disease. Forty-nine patients (38.0%) had cervical dystonia documentation. The cohort was referral-based and pain-enriched by design.

### 3.2 Documented pain phenotypes

Phenotype-codable pain documentation was identified in 125 of 129 patients (96.9%). This proportion reflects successful extraction of a specific pain phenotype among patients already referred because of pain and must not be interpreted as pain prevalence in dystonia.

The most frequent documented phenotypes were limb/extremity pain (94/129, 72.9%), spine/radicular pain (93/129, 72.1%), cervico-occipital/cervicogenic pain (69/129, 53.5%), neuropathic descriptors (56/129, 43.4%), and myofascial/muscle pain (48/129, 37.2%). Widespread/multifocal pain and headache/migraine were each documented in 25 patients (19.4%), and orofacial/trigeminal pain in 18 (14.0%). Phenotypes were non-mutually exclusive.

### 3.3 Documented treatment and procedure exposures

At least one prespecified interventional pain-procedure category was documented in 86 patients (66.7%). Facet or medial branch procedures were documented in 46 (35.7%), epidural or transforaminal epidural steroid injections in 42 (32.6%), radiofrequency or pulsed radiofrequency procedures in 40 (31.0%), and occipital nerve blocks in 14 (10.9%). Botulinum toxin was documented in 49 patients (38.0%) and DBS or other neurosurgical dystonia treatment in 15 (11.6%). Gabapentinoids were documented in 58 patients (45.0%), opioids in 42 (32.6%), and medical cannabis in 40 (31.0%). These findings describe EHR exposure or discussion and not confirmed efficacy.

### 3.4 Prespecified cervical dystonia comparison

Cervico-occipital/cervicogenic pain was more frequently documented among patients with cervical dystonia than among those without cervical dystonia (34/49 [69.4%] vs 35/80 [43.8%]; OR 2.91, 95% CI 1.38–6.18; *P* = .006). When this comparison was included in the broader exploratory multiplicity screen, the Benjamini-Hochberg q value was 0.112.

### 3.5 Exploratory treatment co-documentation

After Benjamini-Hochberg correction, botulinum toxin documentation co-occurred with myofascial/muscle pain (OR 5.44, 95% CI 2.50–11.84; q = 0.0012), cervico-occipital/cervicogenic pain (OR 5.46, 95% CI 2.43–12.25; q = 0.0012), and medical cannabis documentation (OR 3.84, 95% CI 1.75–8.41; q = 0.029). These associations are consistent with treatment indication and clinical complexity and should not be interpreted as biological or therapeutic effects.

## 4 Discussion

This referral-based cohort demonstrates that documented pain among patients with dystonia seen in tertiary pain practice is heterogeneous and frequently accompanied by procedural and medication exposure. The principal contribution is a patient-level description of overlapping anatomical and mechanistic pain features, not an estimate of pain prevalence and not evidence that the documented treatments were effective.

### 4.1 Interpretation relative to the Dystonia-PCS

The Dystonia-PCS offers a validated prospective framework for determining whether pain is directly related to dystonia, aggravated by dystonia, unrelated, or undetermined, while also quantifying burden.5 Our retrospective extraction addresses a different question: which pain concepts and treatment exposures are documented when patients with dystonia reach tertiary pain care. The two approaches are complementary, but retrospective text flags cannot substitute for prospective Dystonia-PCS scoring or standardized pain-intensity and interference measures.

The referral pathway is central to interpretation. Patients entered the cohort because pain had already become clinically important enough to prompt pain-service referral. The high frequency of extracted phenotypes therefore reflects the composition of this selected pathway rather than the frequency of pain among all patients with dystonia.

### 4.2 Phenotype-specific findings

The prespecified association between cervical dystonia and cervico-occipital pain is clinically plausible and consistent with prior literature on painful cervical dystonia.6–9 Potential contributors include dystonic muscle contraction, altered cervical loading, myofascial pain, facet-mediated pain, and occipital nerve irritation. Spine/radicular and limb phenotypes were also common, emphasizing that pain in a patient with dystonia should not automatically be attributed to dystonia. Structural spine disease, radiculopathy, and peripheral nerve disorders require independent clinical evaluation.^15^.

### 4.3 Treatment exposure and indication bias

The substantial documentation of spine-directed procedures, botulinum toxin, gabapentinoids, opioids, cannabis, and DBS indicates high clinical treatment exposure within this cohort. It does not demonstrate refractory pain, confirmed treatment administration, or response. Associations between botulinum toxin and cervico-occipital or myofascial documentation are especially vulnerable to indication bias because the same clinical phenotype may determine both the treatment and the recorded pain terminology. DBS co-documentation may similarly reflect motor severity, disease duration, or referral complexity rather than a pain-specific biological relationship.

### 4.4 Clinical implications

The findings support a structured clinical assessment that separates cervico-occipital/myofascial pain potentially related to dystonia from structural spine/radicular pain, neuropathic-feature pain, widespread pain, and craniofacial pain. This framework may help organize diagnostic evaluation and prospective data collection; the present study did not test whether phenotyping changes procedural selection, reduces overtreatment, or improves outcomes.

### 4.5 Limitations

The study has several limitations. First, referral-based enrichment limits generalizability and precludes prevalence estimates. Second, the retrospective design relies on variable clinical documentation and lacks standardized pain scores, Dystonia-PCS ratings, complete dystonia subtype and etiology, and validated motor-severity measures. Third, the NLP pipeline identifies documentation patterns rather than adjudicated diagnoses. Although a blinded manual audit of 129 category-stratified random records was performed, record-level contingency tables were not retained; formal sensitivity, specificity, positive predictive value, and inter-rater agreement could therefore not be reported. A single senior physician performed the audit, precluding inter-rater reliability assessment.

Fourth, treatment flags encompass administration, prescription, planning, or discussion and do not establish duration, indication, adherence, or efficacy. Fifth, associations were unadjusted and vulnerable to indication bias and residual confounding. The modest cohort size and incomplete covariate data prevented reliable multivariable adjustment. Finally, aggregation across a long observation period does not establish temporal ordering between pain phenotypes and treatments.

### 4.6 Future directions

Prospective studies should apply the Dystonia-PCS together with anatomical pain maps, Brief Pain Inventory measures, DN4 or painDETECT screening, motor-severity scales, dystonia subtype and etiology, and treatment timing. A formally validated multilingual extraction pipeline should retain record-level reference labels and report sensitivity, specificity, predictive values, F1 score, and inter-rater agreement. These data would permit testing whether structured phenotyping improves treatment selection and patient-centered outcomes.

## 5 Conclusions

Among 129 patients with dystonia referred to tertiary pain care, documented pain phenotypes were heterogeneous and frequently accompanied by interventional and medication exposure. Cervical dystonia was associated with cervico-occipital pain, while spine/radicular and limb phenotypes were also prominent. The results support structured prospective pain assessment but do not establish population prevalence, adjudicated diagnoses, causal associations, or treatment efficacy.

## CRediT authorship contribution statement

**Shachar Zion Shemesh:** Formal analysis, Data curation. **Tsvia Fay-Karmon:** Writing – review & editing. **Paz Kelmer:** Writing – review & editing, Writing – original draft. **Jose Asprilla:** Validation. **Zaki Hinnawi:** Project administration. **Itay Goor-Aryeh:** Supervision. **Yotam Hadari:** Project administration. **Lior Ungar:** Supervision.

## Ethics approval

The study was approved by the Institutional Review Board/Helsinki Committee of Sheba Medical Center, Tel Hashomer, Israel (approval no. SMC-9054-22). The requirement for informed consent was waived because the study involved retrospective analysis of de-identified clinical data.

## Funding

This research received no external funding.

## Declaration of competing interest

The authors declare that they have no known competing financial interests or personal relationships that could have appeared to influence the work reported in this manuscript. All authors confirm that there are no conflicts of interest relevant to this study.

## Data Availability

De-identified analytic outputs may be made available from the corresponding author upon reasonable request and subject to institutional approval. Original free-text EHR data cannot be publicly shared because they contain protected clinical information.
